# Importance of Serum Amino Acid Profile for Induction of Hepatic Steatosis under Protein Malnutrition

**DOI:** 10.1038/s41598-018-23640-8

**Published:** 2018-04-03

**Authors:** Hiroki Nishi, Daisuke Yamanaka, Hiroyasu Kamei, Yuki Goda, Mikako Kumano, Yuka Toyoshima, Asako Takenaka, Masato Masuda, Yasushi Nakabayashi, Ryuji Shioya, Naoyuki Kataoka, Fumihiko Hakuno, Shin-Ichiro Takahashi

**Affiliations:** 1 Departments of Animal Sciences and Applied Biological Chemistry, Graduate School of Agriculture and Life Sciences, The University of Tokyo, Tokyo, Japan; 2 Department of Veterinary Medical Sciences, Graduate School of Agriculture and Life Sciences, The University of Tokyo, Tokyo, Japan; 3 Department of Bioregulation, Nippon Medical School, Kanagawa, Japan; 4 Department of Agricultural Chemistry, School of Agriculture, Meiji University, Kanagawa, Japan; 5 Center for Computational Mechanics Research, Toyo University, Kawagoe, Japan

## Abstract

We previously reported that a low-protein diet caused animals to develop fatty liver containing a high level of triglycerides (TG), similar to the human nutritional disorder “kwashiorkor”. To investigate the underlying mechanisms, we cultured hepatocytes in amino acid-sufficient or deficient medium. Surprisingly, the intracellular TG level was increased by amino acid deficiency without addition of any lipids or hormones, accompanied by enhanced lipid synthesis, indicating that hepatocytes themselves monitored the extracellular amino acid concentrations to induce lipid accumulation in a cell-autonomous manner. We then confirmed that a low-amino acid diet also resulted in the development of fatty liver, and supplementation of the low-amino acid diet with glutamic acid to compensate the loss of nitrogen source did not completely suppress the hepatic TG accumulation. Only a dietary arginine or threonine deficiency was sufficient to induce hepatic TG accumulation. However, supplementation of a low-amino acid diet with arginine or threonine failed to reverse it. *In silico* analysis succeeded in predicting liver TG level from the serum amino acid profile. Based on these results, we conclude that dietary amino acid composition dynamically affects the serum amino acid profile, which is sensed by hepatocytes and lipid synthesis was activated cell-autonomously, leading to hepatic steatosis.

## Introduction

Nutrients are one of the most fundamentally important external elements for all organisms to maintain their health and reproductive performance. There exist many kinds of nutrient-monitoring systems across species, from yeast to human, in order to properly sense the nutritional environment in which they live^1^. In most cases, when the shortage/excess of each nutrient is detected in each tissue, a specific signaling pathway is activated, which then evokes a responsive metabolic reaction^1^.

Protein is one of the most important nutrients because nearly half of the dry weight of a mammalian body is made of proteins^2^ which have incredibly diverse biological functions. Besides, some amino acids are called essential amino acids due to the weak biosynthetic activity and thus the necessity for enough dietary intake. It, therefore, is quite significant to monitor proteins or amino acids as nutrients, consistent with the fact that the amino acid-monitoring system is highly conserved among many species^1^.

In recent years, many reports have revealed that amino acids are not only passive nutrients or building blocks of proteins, but are also active signaling molecules^3^. When the presence or shortage of amino acids is sensed by the monitoring system, specific signaling pathways are activated, resulting in the specific physiological responses. For example, amino acids directly stimulate the hypothalamus leading to the regulation of satiety or feeding behavior^4^, and amino acid-deprivation induces secretion of fibroblast growth factor (FGF) 21, a hormone essential for adapting to starvation, from the liver in a general control nonderepressible (GCN) 2-activating transcription factor (ATF) 4-dependent manner^3^. Furthermore, some recently discovered cytosolic amino acid sensors link the amino acid signals and mammalian target of rapamycin complex (mTORC) 1 signaling^5,6^. These results support the concept that amino acids function as endocrine factors extracellularly and as signal-mediating factors intracellularly, to regulate the metabolism.

We previously demonstrated that growing rats fed a low-protein diet showed reduced serum insulin and insulin-like growth factor (IGF)-I levels, followed by mild growth retardation^7–9^ and they also showed improved insulin sensitivity^10^. Additionally, we also discovered that in such animals, triglyceride (TG) accumulated in their livers, forming fatty livers^9^. We hypothesized that increased sensitivity to insulin may be one of the causes of fatty liver development; however, we also obtained data indicating TG accumulation in the liver is independent of the upregulation of insulin signaling^11^. This phenomenon is also known among human disorders referred to as *kwashiorkor*, related to protein malnutrition with representative symptoms of edema of limbs, growth retardation and hepatomegaly resulting from hepatosteatosis^12^. These results suggest that dietary proteins or amino acid signaling can affect the metabolic regulation, leading to a non-alcoholic fatty liver disease (NAFLD)-like phenotype.

NAFLD is a worldwide emerging concern in association with an epidemic of diabetes and related disorders. There are known to be many kinds of factors leading to NAFLD, but mechanisms to develop the hepatic steatosis remain largely to be elucidating^13^. In our current study, we focused on the fatty liver formation attributed to amino acid deficiency. Our results revealed new insights into how a change in dietary amino acid compositions or amino acid signaling induces lipid accumulation in the liver or hepatocytes.

## Results

### Effects of amino acid deficiency on TG accumulation in hepatocytes

**Figure 1:**
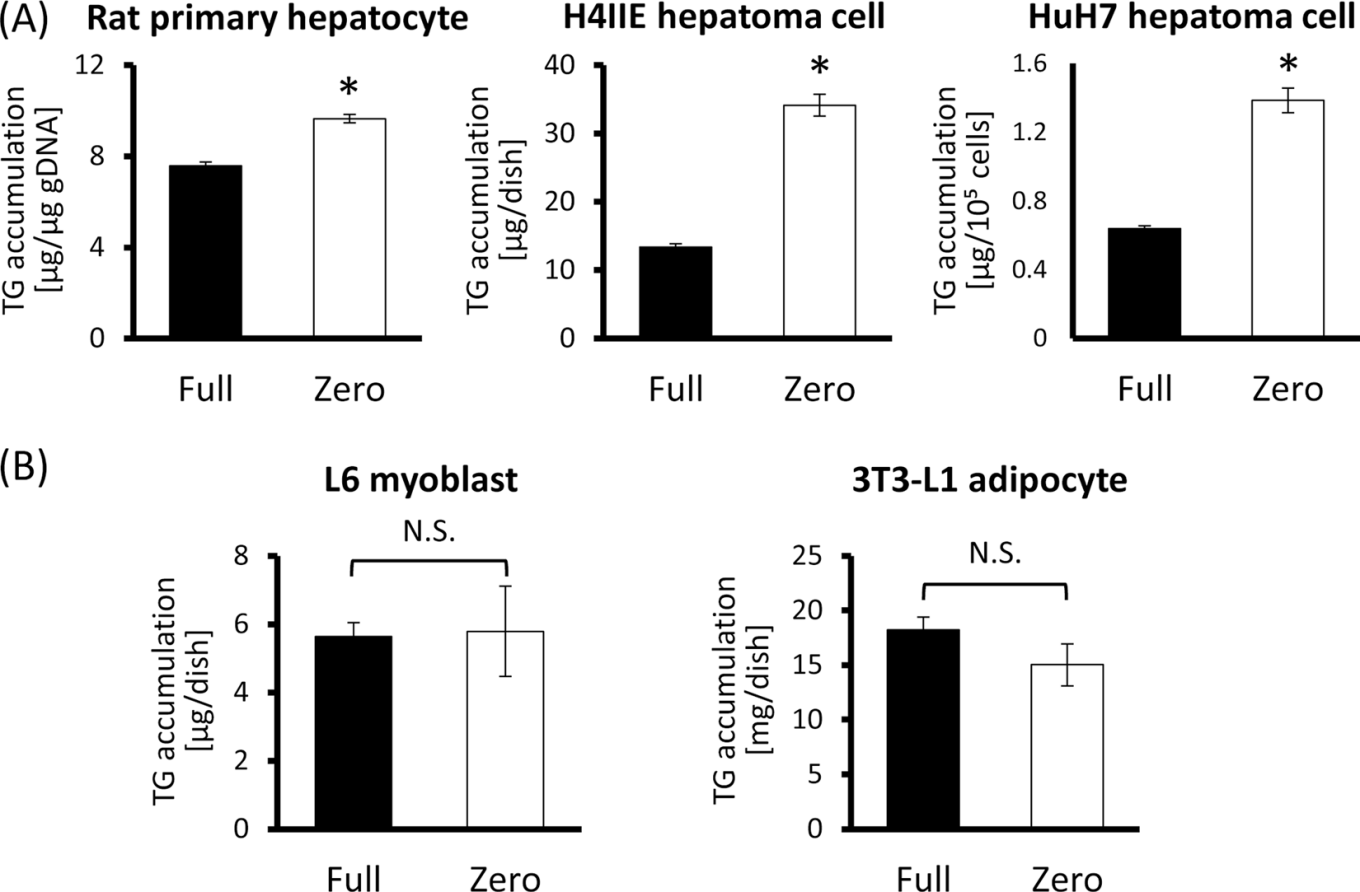
TG accumulation is induced in hepatocytes cell-autonomously in response to amino acid-deficiency. (**A**) Hepatocytes (rat primary hepatocytes, H4IIE rat hepatoma cells and HuH7 human hepatoma cells) were cultured for 24 hours in a medium containing 20 amino acids sufficiently (Full) or no amino acids (Zero), and then intracellular TG levels were measured. (**B**) H4IIE cells were cultured for 24 hours in Full, Zero medium or media lacking only a single amino acid and then intracellular TG levels were measured. bar; means ± S.E. (n = 3), *p < 0.05 (A, Student’s t-test; B, Tukey-Kramer’s test vs Full).

Given the previous results, we hypothesized that “serum” amino acids may have immediate influence on the induction of TG accumulation in the liver. To examine this hypothesis, we utilized a cell-culture model in which hepatocytes were cultured in complete medium (Full) or amino acid-deprived medium (Zero) (See Supplementary Table 1). As a hepatocyte model, we used HuH7 and H4IIE cells, human and rat hepatoma cell lines respectively, and rat primary hepatocytes. After a 24-hour-culture in each medium, the TG levels in the cells were measured. When the cells were cultured in Zero medium, TG accumulation was accelerated in every cell line, compared to those cultured in Full medium (Fig. 1A, primary hepatocytes, *p* = 0.0028; H4IIE, *p* = 0.0005; HuH7, *p* = 0.0012). In addition, using L6 rat myoblasts and 3T3-L1 mouse adipocytes, such tendency to increase the cytosolic TG in Zero medium was not observed (Fig. 1B). Interestingly, only TG level was increased in H4IIE cells while another neutral lipid, cholesterol was not (Supplementary Fig. S1A). These results suggested that hepatocytes have the ability to induce the intracellular TG accumulation cell-autonomously in response to amino acid deficiency, which is likely to be specific for hepatocytes.

### Effects of amino acid deficiency on glucose-dependent *de novo* lipid synthesis in hepatocytes

**Figure 2:**
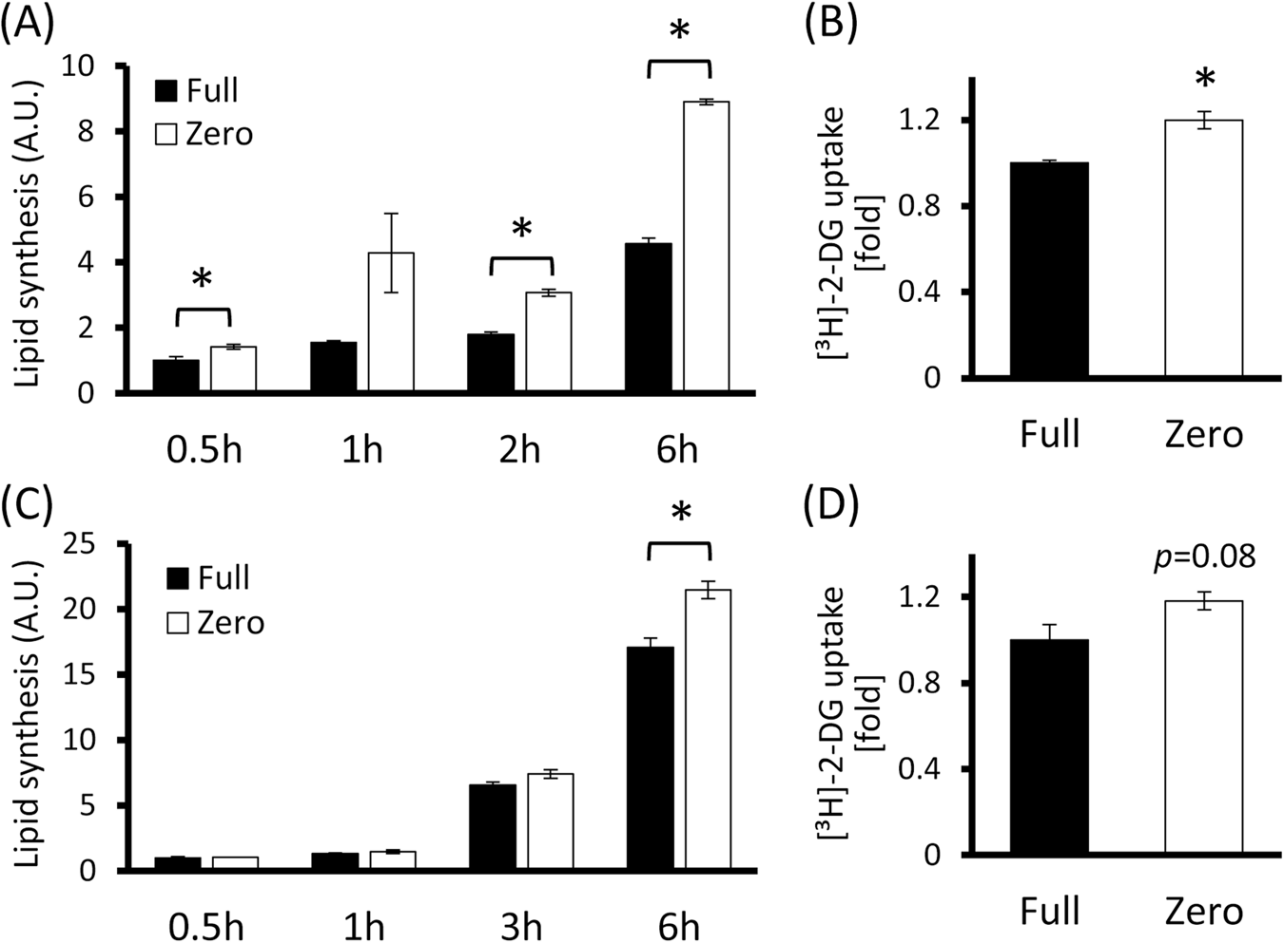
Amino acid-deficiency causes hepatocytes to enhance *de novo* lipid synthesis derived from glucose. (**A**,**B**) H4IIE cells (**A**) and HuH7 cells (**B**) were cultured in Full or Zero medium containing [14C]-labeled glucose for indicated hours and total lipids were extracted with methanol/chloroform. Lipid synthesis was quantified by measuring the radioactivity in the lipids. (**C**,**D**) Glucose uptakes of H4IIE cells (**C**) and HuH7 cells (**D**) were measured using [³H]-labeled 2-deoxy-D-glucose (2-DG) which is a glycolysis-resistant analogue of D-glucose. After 6-hour pre-culture in serum- and glucose-free medium, the medium was changed to Full or Zero medium containing [³H]-2-DG and incubated for 30 minutes.

In order to examine the detailed mechanisms for this TG accumulation, we analyzed the lipid synthesis activity. H4IIE cells were cultured in Full or Zero medium containing [^14^C]-labeled D-glucose, then lipids in their lysates were extracted by bilayer separation and ^14^C radioactivities in the organic layer were measured. The results indicated that lipid synthesis was elevated when the cells were cultured in Zero medium compared to Full medium (Fig. 2A, P-values of Full vs Zero at 0.5, 1, 2 and 6-hour were 0. 0319, 0.0978, 0.0001 and <0.0001 respectively), showing the conversion of glucose into lipids was activated in response to amino acid deficiency. Supporting this, glucose uptake was also slightly increased in Zero medium (Fig. 2B, *p* = 0.0171), and moreover, HuH7 cells showed the same tendency as H4IIE cells (Fig. 2C, P-values of Full vs Zero at 0.5, 1, 2 and 6-hour were 0.7294, 0.3817, 0.1182 and 0.0073 respectively; Fig. 2D, *p* = 0.089).

### Effects of amino acid pool and insulin on TG accumulation in hepatocytes

**Figure 3:**
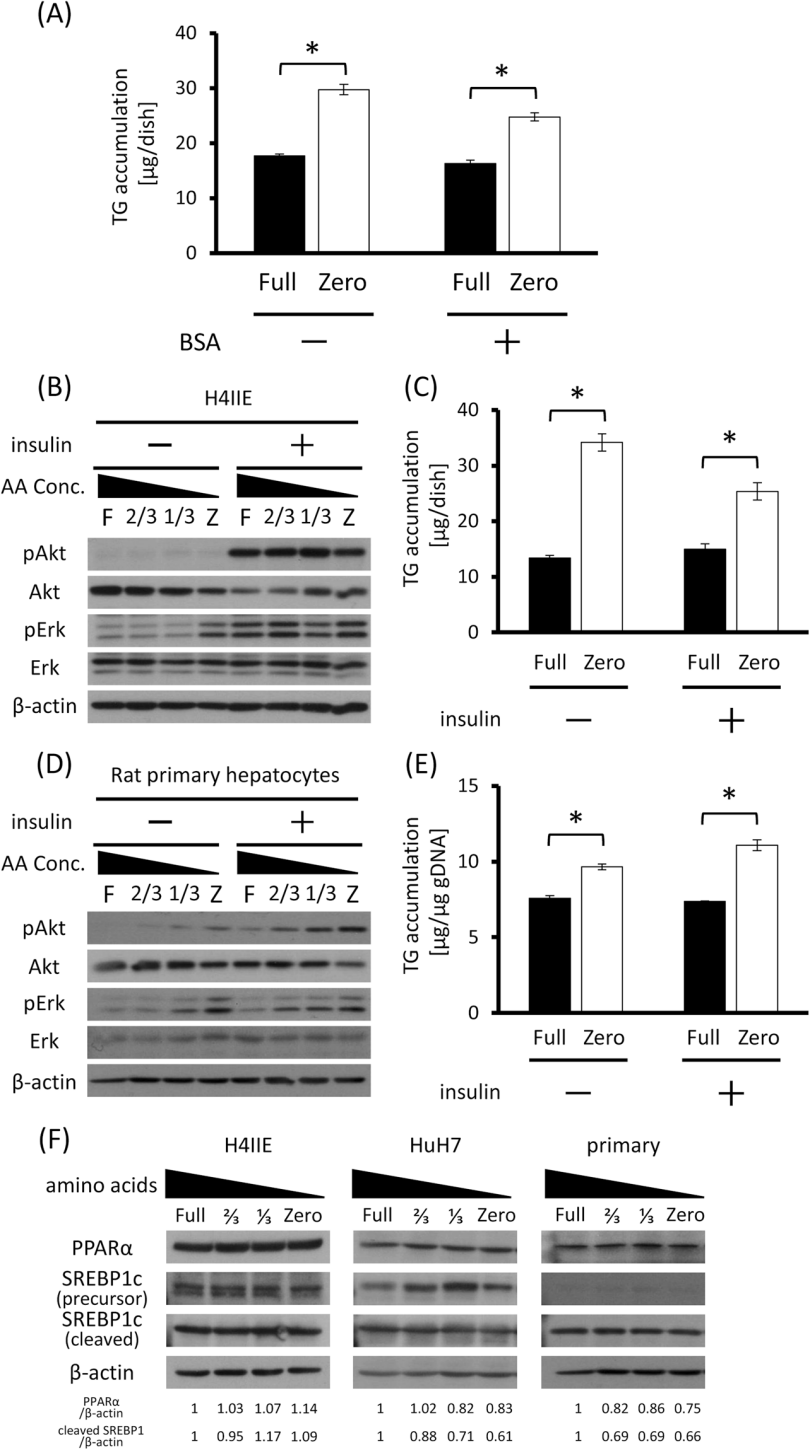
TG accumulation in hepatocytes is driven by amino acid pool, independently of insulin and major lipid metabolism-related transcription factors. (**A**) H4IIE cells were cultured in Full or Zero medium with or without 1.38 g/L bovine serum albumin (BSA) and TG content in the cells were measured. (**B**–**E**) H4IIE cells (**B**,**C**) and rat primary hepatocytes (**D**,**E**) were incubated in Full or Zero medium with or without 10 nM insulin for 24 hours. Phosphorylation of Akt and Erk, which are major signaling proteins mediating insulin signaling cascade, was detected by immunoblotting (**B**,**D**) and TG content in cells was measured (**C**,**E**). (**F**) The expression levels of PPARα and SREBP1c in H4IIE (left), HuH7 (middle) and rat primary hepatocytes (right) were detected by immunoblotting. Density of each band was quantified using ImageJ software and shown under the blot image bar; means ± S.E. (n = 3), *p < 0.05 (Student’s t-test).

Our cell culture model using Full and Zero medium was a simple model containing no serum or proteins in the media. However, in the physiological environment surrounding the liver/hepatocytes, there is a vast reservoir of proteins such as albumin and hormones. Thus, to test whether the extracellular proteins can affect amino acid deficiency-induced TG accumulation, we cultured H4IIE cells in Full or Zero medium containing bovine serum albumin (BSA). TG accumulation still increased in Zero medium regardless of the presence of BSA (Fig. 3A, Full vs Zero <BSA−>, *p* = 0.0006; Full vs Zero <BSA+>, *p* = 0.0019), indicating that in the context on TG accumulation, H4IIE cells sense the deficiency in “free”, but not protein-formed, amino acids.

We next examined the effect of insulin on amino acid deficiency-induced TG accumulation. Insulin is a major anabolic hormone which is generally believed to stimulate lipid anabolism. We added insulin to Full and Zero medium while culturing H4IIE cells, and then measured the intracellular TG levels. Interestingly, TG accumulation in H4IIE cells with insulin stimulation was not significantly different from that in insulin-free conditions, whereas downstream insulin signaling pathways were indeed activated (Fig. 3B,C, Full vs Zero <insulin−>, *p* = 0.0005; Full vs Zero <insulin+>, *p* = 0.0098). Similar results were observed using rat primary hepatocytes (Fig. 3D,E, Full vs Zero <insulin−>, *p* = 0.0028; Full vs Zero <insulin+>, *p* = 0.0011). Taken together, these results demonstrated that “free” amino acid deficiency stimulates hepatocytes to accumulate TG, which is accompanied by increased *de novo* lipid synthesis from glucose.

Peroxisome proliferator-activated receptor α (PPARα) and sterol regulatory element-binding protein-1c (SREBP1c) are generally considered to be important transcription factors for the regulation of hepatic lipid metabolism. We then measured the expression levels of PPARα or SREBP1c in each type of hepatocyte, but their expression levels were not significantly changed by culture in Zero medium (Fig. 3F), suggesting that mechanisms conventionally considered to be important to regulate hepatic lipid metabolism could be less involved in this TG accumulation in response to amino acid deficiency.

### Effects of each amino acid deficiency on TG accumulation in hepatocytes

**Figure 4:**
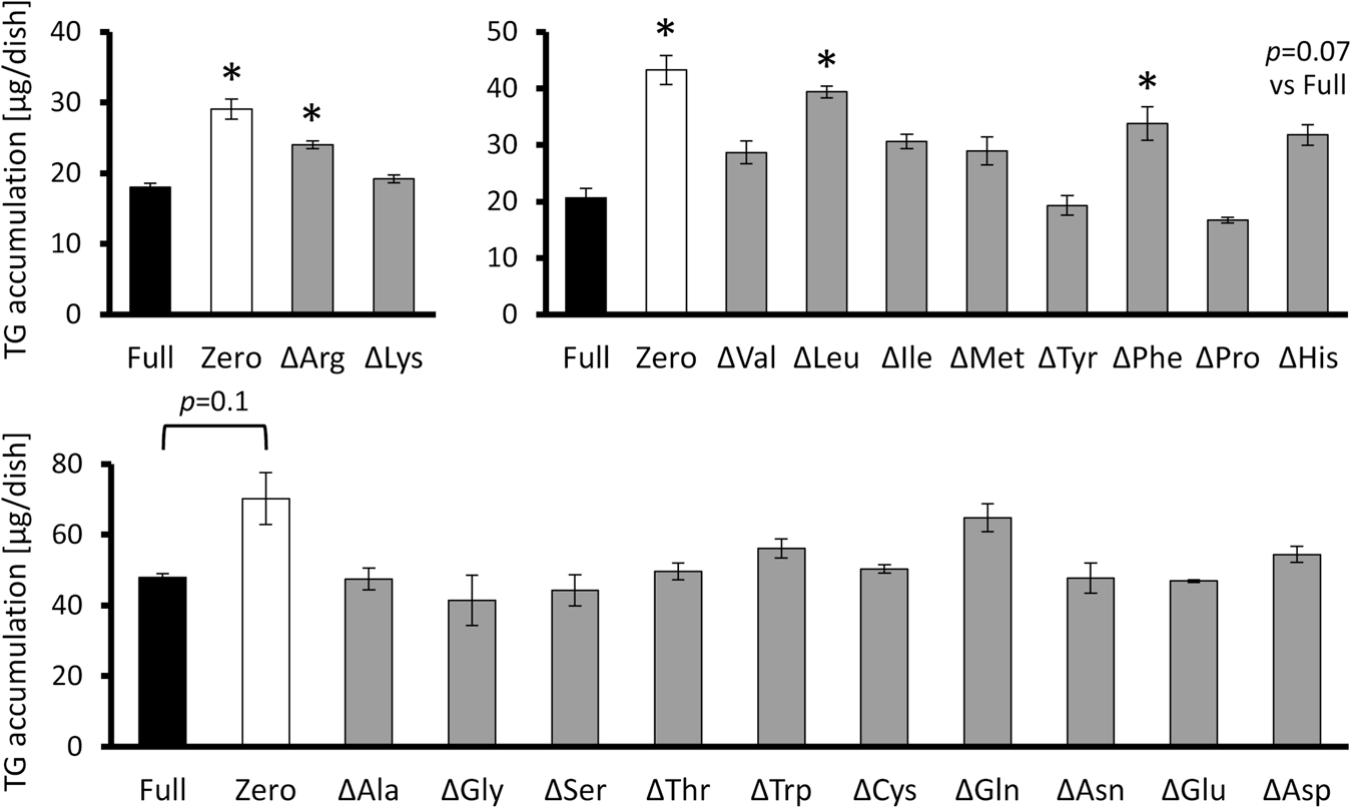
Each amino acid can have different roles in amino acid deprivation-induced TG accumulation in hepatocytes. H4IIE cells were cultured for 24 hours in Full, Zero medium or media lacking only a single amino acid and then intracellular TG levels were measured means ± S.E. (n = 3), *p < 0.05 vs Full.

In order to know the deprivation of which amino acid out of 20 major amino acids has the largest impact on amino acid deprivation-induced lipid accumulation, we prepared single amino acid-deprived media (Δamino acid medium, see Supplementary Table 1). When H4IIE cells were cultured in each medium for 24 hours, some single amino acid deprivations (ΔArg, ΔLeu, ΔPhe and ΔHis) also enhanced (or tended to enhance) intracellular TG accumulation, though it was not as strongly as Zero medium (Fig. 4). But in contrast, supplementation of these four amino acids into Zero medium did not reverse TG accumulation driven in Zero medium (Supplementary Fig. S2). These implied that each amino acid has a different role in the induction of TG accumulation in hepatocytes, and not only the total amino acid amount but also the amino acid composition in the medium was important for lipid metabolic regulation in hepatocytes.

### Effects of each dietary amino acid deficiency on TG accumulation in the liver

**Figure 5:**
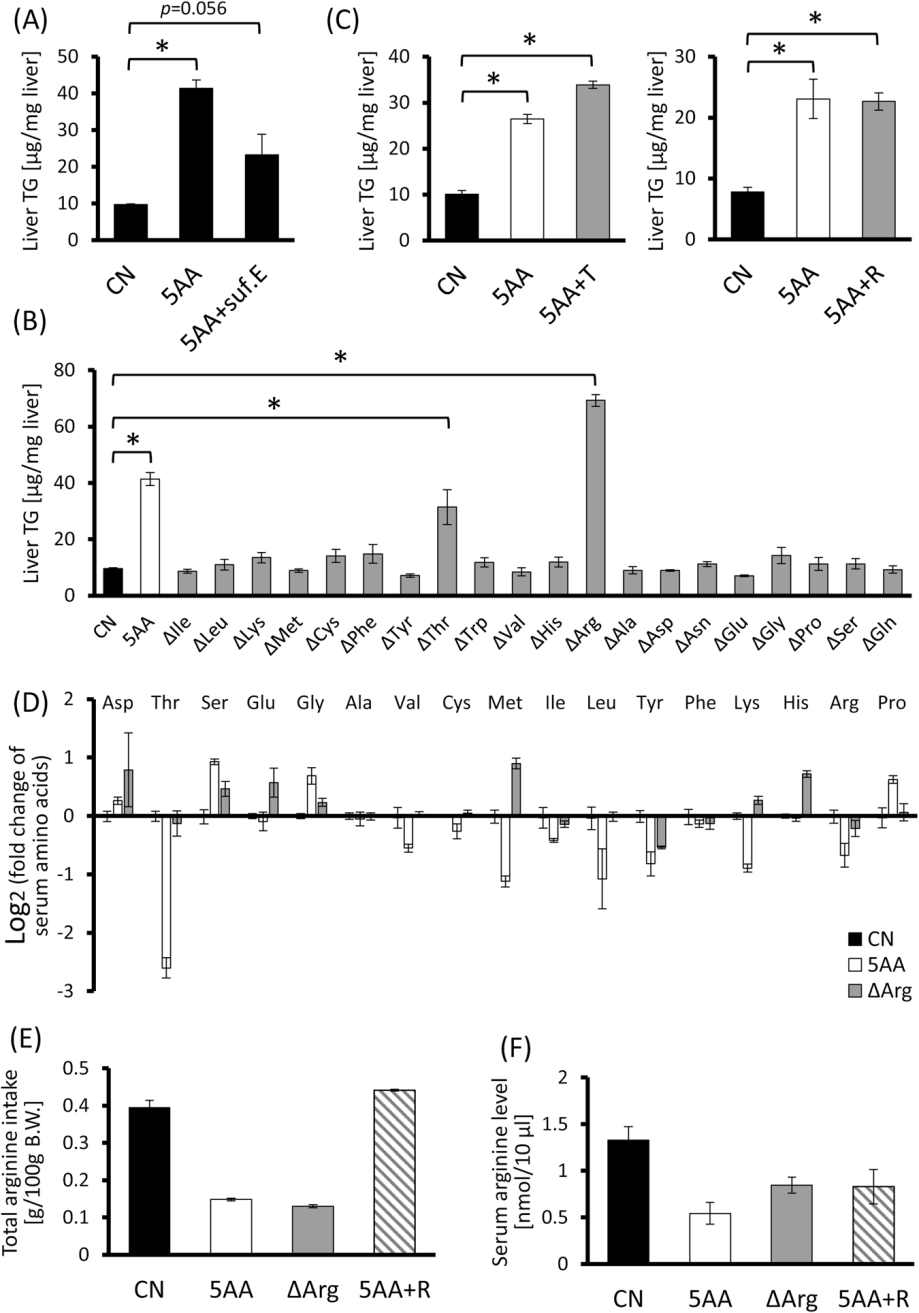
Dietary arginine-deficiency causes hepatic TG-accumulation, but it does not sufficiently explain total amino acid-deficiency-induced fatty liver formation. Six-week-old male Wistar rats were fed experimental diets ad libitum for seven days and their livers and bloods were collected. (**A**) TG levels in the liver of the rats fed a control diet (CN), a low-amino acid diet (5AA) or a glutamic acid-supplemented 5AA diet in which the total amount of amino acids was comparable to CN were measured. (**B**) TG levels in the liver of the rats fed diets deficient in a single amino acid. (**C**) TG levels in the liver of the rats fed a 5AA diet, or threonine- (5AA + T) or arginine- (5AA + R) supplemented 5AA diet. (**D**) Relative serum amino acid concentrations of the rats fed a 5AA and a ΔArg diets were analyzed. (**E**,**F**) The total amounts of arginine intake (**F**) and serum arginine concentrations (**G**) of the rats fed a 5AA, a ΔArg or a 5AA + R diet were measured. bar; means ± S.E. (n = 3–4), *p < 0.05 (Tukey-Kramer’s test).

We have previously shown that the fatty liver could be induced by low-protein diet^9^. In order to manipulate the dietary amino acid compositions, we prepared here experimental diets containing sufficient (15%, w/w) amino acid mixture (control nutrition; CN) or 5% (w/w) amino acids (5 AA) (see Supplementary Table 2), and fed them to 6-week-old male Wistar rats for one week. As low-protein diet did, 5 AA diet also caused significant TG accumulation in the liver (Fig. 5A, *p* = 0.0048), while cholesterol level was just slightly increased, consistent with cell-culture model (Supplementary Fig. S1B). Additionally, supplementation of the 5AA diet with glutamic acid to compensate for the loss of nitrogen source did not completely diminish the TG accumulation in the liver (Fig. 5A), implying that the shortage of nitrogen source was not a critical reason for the TG accumulation in response to dietary amino acid-deficiency. We then prepared the diets deficient in each single amino acid in which the amount of only a single amino acid was reduced to the same as that of a 5AA diet and the others were the same as a CN diet (Δamino acid diet, see Supplementary Table 2). As a result, a threonine- or an arginine-deficient diet could induce hepatic TG accumulation (Fig. 5B, P-values of 5AA, ΔThr and ΔArg vs CN were all <0.0001). Remarkably the extent of TG-content in ΔArg group was much greater than a 5AA diet group. So to investigate whether lack of dietary threonine or arginine intake was the cause for 5AA diet-induced TG accumulation, as much threonine or arginine as a CN diet was added to a 5AA diet (5AA + T, 5AA + R). To our surprise, however, TG levels in the liver were comparable between a 5AA and a 5AA + T or 5AA + R group (Fig. 5C, P-values of 5AA and 5AA + T against CN were <0.0001, and P-values of 5AA and 5AA + R against CN were 0.0037 and 0.0045 respectively). These results suggested that hepatic TG accumulation caused by dietary amino acid deficiency could not be explained in terms of just a total amount of single amino acid intake.

### The relationship between dietary amino acid composition and serum amino acid profile

We next focused on serum amino acid concentrations, since our present data illustrated that hepatocytes could cell-autonomously respond to a change in extracellular amino acid concentrations. 5AA or ΔArg diet-feeding actually modified the serum amino acid profiles of rats dynamically, but remarkably, they did not necessarily reflect the dietary amino acid compositions (Fig. 5D). In the serum of 5AA group, several essential amino acid levels were decreased while the concentrations of serine and glycine were considerably increased. On the other hand, the ΔArg group showed high levels of serum glutamic acid, methionine and histidine, while the tyrosine level was decreased. Especially, though dietary arginine intake of 5AA and ΔArg groups were almost the same (Fig. 5E), the serum arginine level of a ΔArg group was higher than a 5AA group (Fig. 5F). And though arginine intake of a 5AA + R group was much higher than a ΔArg group (Fig. 5E), their serum arginine levels were comparable (Fig. 5F). These results showed that serum amino acid profile could change in a very complexed manner, apparently not consistent with dietary amino acid composition, and therefore it was also hard to expect the influence of dietary amino acids on a serum amino acid profile.

### Hepatic TG accumulation level *in vivo* can be estimated by means of a serum amino acid profile

To examine the relationship between serum amino acids and hepatic lipid metabolism, we analyzed the correlation between serum amino acid concentrations and the TG accumulation level in the liver. But as mentioned above, we did not find any correlation in terms of a single amino acid (Fig. 4). Thus we dealt with individual data sets of serum amino acid concentrations as multi-dimensional vector data to conduct a comprehensive *in silico* analysis using machine learning programs. As an input data, we took data from a total of 135 rats which were fed CN diet, 5AA diet, Δamino acid diets or 5AA diets supplemented with 20 kinds of single amino acids. In all, 90 data sets were used for a training and 45 data sets were for a test.

**Figure 6:**
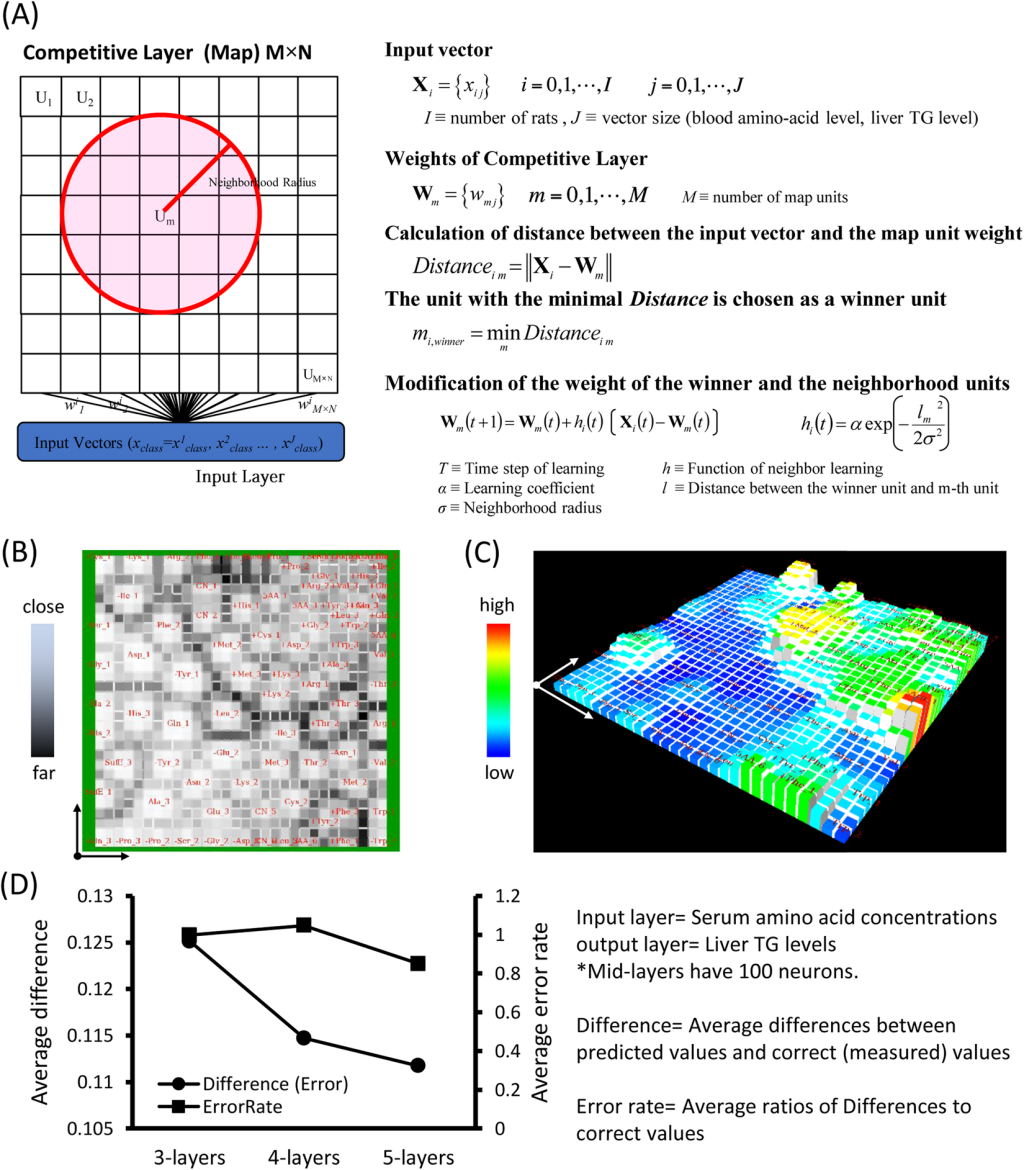
Liver TG level can be estimated from serum amino acid profile by machine learning programs. (**A**) A schematic illustration about SOM learning and algorithm. (**B**) The map obtained using serum amino acid concentrations of each rat as input vectors. Similar vectors were put near forming some clusters. Color represents the distance between neighborhood vectors. (**C**) Bars are placed on the map obtained in (**A**) and the height represents measured TG levels in the liver of each rat. (**D**) A comparison of prediction accuracies of liver TG levels using serum amino acid concentrations as input vectors, obtained by MLPs which have the different number of mid-layers. In both SOM and MLP experiments, data size was 135 (Data sets of serum amino acid concentrations and a liver TG level were obtained from 135 rats.). Out of that, 90 were for learning and 45 were for test.

Self-Organizing Map (SOM), one of the unsupervised machine learning programs^14,15^, can classify high-dimensional data sets, putting them in a lower-dimensional space (usually two- or three-dimensional) according to their similarity to each other (Fig. 6A). On the map, “similar” data sets will be put near. We have already reported that we could smoothly classify rats by SOM, using individual serum amino acid concentrations and hepatic TG level as input vectors^16^. Here, we carried out the SOM analysis using only serum amino acid concentrations as input data which did not include the data on liver TG. By this analysis, we obtained a 30 × 30 square map on which rats with similar serum amino acid profiles were successfully put near each other (Fig. 6B). Then liver TG levels of each rat were shown on the map as bars (Fig. 6C). Even though the classification was made only based on the data on serum amino acid concentrations, liver TG level was also smoothly classified, suggesting that the liver TG level was correlated well with a serum amino acid profile. In addition, we put on the map several unlearned individual data of only serum amino acid concentrations, which had not been used as input data to make the map. As a result, the liver TG value of the cell on which the “unlearned” vector was mapped, was similar to the actual liver TG level of the “unlearned” individual (Table 1), suggesting again that comprehensive serum amino acid profile correlated well to hepatic TG level.

**Table 1:** Estimation of liver TG levels by serum amino acid profiles using SOM.

	Estimated TG	Correct TG	Error Rate
CN #1	0.043697	0	—
CN #2	0.169301	0.08186	1.068177
5AA #1	0.424717	0.320658	0.324517
5AA #2	0.587921	0.548892	0.071105

The “unlearned” rats were located on the map obtained in Fig. 4A and liver TG levels of them were estimated by their relative positions on the map. Estimated liver TG levels and measured liver TG levels of each “unknown” rat were compared. Average difference = 0.111737. Average error rate = 0.850083. *All values are standardized from 0 to 1.

Next, we applied another machine learning algorithm, multi-layer perceptron (MLP), which belongs to Neural Network. SOM is an unsupervised algorithm for just a classification or categorization of input vectors, while MLP is a supervised one which can estimate the mathematical relationships between input and output vectors^17^. We took serum amino acid concentrations of each rat as input data, and liver TG as output data to conduct MLP analysis. For learning, we made several MLPs whose numbers of mid-layers were 3, 4 and 5. After learning was completed, the serum amino acid concentrations of “unlearned” rats were put into each algorithm to obtain their output liver TG values. Then the obtained liver TG values were compared with measured actual liver TG values, and differences and error rates were calculated. As a result, higher number of mid-layers exhibited a lower error rate and individual hepatic TG accumulation levels could be predicted using just a data set of serum amino acid concentrations with good accuracy (Fig. 6D).

## Discussion

The liver is the largest metabolic organ and many pathways are known to regulate energy metabolism in the liver, including humoral and neuronal inter-organ crosstalk^3,18,19^. Organs such as the brain and the gut can sense the ingested nutrients and transmit the signals for other organs to maintain the energy homeostasis^3,20^. Proteins or amino acids as nutrients are also known to be sensed by such organs/tissues, which then stimulate cross-organ communication, leading to the metabolic regulation in the liver^3,10,21^. For instance, rats with dietary amino acid deficiency show a decrease in insulin secretion and they exhibit increased insulin sensitivity and lower gluconeogenic activity^10^. We therefore have long assumed that amino acid deficiency-induced fatty liver development was caused by the change in the endocrinological environment, particularly the modulation of insulin-like activities. However, our recent study showed that restoration of serum insulin to a low-protein diet fed animals by injecting insulin does not affect hepatic TG accumulation^22^. Our current results demonstrated that hepatocytes responded cell-autonomously to amino acid deprivation or a change in amino acid composition in the medium to induce TG accumulation, independently of insulin action (Figs 1–4). Similarly it was shown that a depletion of amino acid starvation-responsive hormone, FGF21 also does not reverse hepatic TG accumulation in response to amino acid deficiency^23^. These results implied the importance of cell-autonomous regulation by amino acids, apart from hormonal stimulation in the context of hepatic lipid metabolism. This idea is in agreement with some previous reports. For example, the autophagic activity, which is also regulated by both insulin and amino acids, is more strongly affected by amino acids than insulin in the liver, while in skeletal muscle insulin has a stronger effect^24^. Collectively, although the significance of hormonal regulation still cannot be excluded, these facts suggest that amino acids may possibly be the more dominant regulator of some aspects of hepatic metabolism.

On the other hand, we showed *in vivo* that out of 20 protein-constructing amino acids, only a threonine- or arginine-deficiency in a diet could increase the hepatic TG levels in rats (Fig. 5). Actually, the relationships between dietary threonine- or arginine-deficiency and hepatic lipid metabolism have been implied since the late twentieth century^25,26^, but the detailed mechanisms to develop the fatty liver have been largely unknown to date. In the present study, from the view of whether the threonine- or arginine-deficiency was responsible for low-amino acid diet-induced hepatic TG accumulation, we fed a threonine- or arginine-supplemented 5AA diet to rats. However, the supplementation did not affect the TG accumulation in the liver caused by a 5AA diet (Fig. 5C). Similarly, though it is well established for methionine-choline deficient diet (MCD) to induce fatty liver, there is a report showing methionine restriction prevents hepatic steatosis in ob/ob mice^27^ and our result demonstrated only a methionine deficient diet did not induce hepatic TG accumulation (Fig. 5B). These results suggested that, in spite of the significance of dietary amino acid composition, it was difficult to explain the amino acid deficiency-induced fatty liver formation only by the amounts of a single amino acid intake and consequently focused our attention on the serum amino acids.

One remarkable point making it complicated to decipher this mechanism is that the results of a rat model and a cell-culture model were apparently different at a glance. For instance, dietary threonine deficiency could induce hepatic TG accumulation, whereas threonine-deficient medium could not as well in H4IIE cells (Figs 4 and 5B). This difference can be expected to result from the dynamic and complicated change in serum amino acid profile in response to a manipulation of dietary amino acid composition. For example, when an animal was deficient in dietary arginine, its serum arginine level did not decrease much while methionine and histidine levels were increased significantly (Fig. 5D). Taken together, we considered such an unexpected change in serum amino acid profile in response to a modulation of dietary amino acid composition to be the key to fill a gap between the results of a rat model and a cell-culture model. And it led us to a working hypothesis that the quality or quantity of dietary proteins or amino acids dynamically affects the serum amino acid profile, which is sensed by the liver, regulating hepatic lipid metabolism as amino acid signaling in a cell-autonomous manner. Since glucagon is a well-documented hormone involved in amino acid metabolism^28^ and the serum glucagon level was slightly lower in 5AA-group than CN-group (Supplementary Fig. S3), it can be possible that serum amino acid profile modulations in response to dietary amino acid composition is mediated by such hormone activities.

Supporting this idea, a mathematical analysis revealed that serum amino acid profiles correlated with liver TG levels enabling us to predict an individual hepatic TG accumulation level using its serum amino acid profile (Fig. 6). This indicates again the serum amino acid is indeed one of the determinant factors for hepatic lipid accumulation. Moreover, not a single amino acid concentration but a comprehensive amino acid profile is more critical. For machine learning analysis, the data size we used was relatively small, but if it is possible to take bigger data such as human clinical data, we should hopefully get a higher prediction accuracy and more reliable estimation. That could consequently lead to a clinical application, such as a new kind of a simple diagnosis for fatty liver just by collecting blood or a simple treatment or prevention method for NAFLD just by modification of dietary amino acid intake.

In recent years, the overview of the amino acid signaling is gradually becoming unveiled. Many solute carrier protein (SLC) amino acid transporters and putative amino acid sensor proteins have been indicated to mediate the amino acid signaling^5,6,29^, in which the signals are usually interpreted as GCN2 signaling^30^ or mTOR signaling^31^. Additionally, in the latest report, another possible amino acid signaling pathway was mentioned, in which a specific amino acid directly binds to some transcriptional regulators in cytosol, causing the structural change and possibly the modulation of their regulatory effects on transcription^32^. While GCN2 is believed to sense the shortage of total amino acids, the other pathways are considered to recognize the specific cytosolic amino acid levels such as leucine, glutamine and arginine^29,32^. Similarly, only threonine- and arginine-deficiency in a diet resulted in development of fatty liver and single deprivation of some specific amino acids induced TG accumulation in hepatocytes (Figs 4 and 5B). Furthermore, it is reported that a lysine-deficient diet drives lipid accumulation in the skeletal muscle in swine^33^. These results indicate that each amino acid may have the specific effects on lipid metabolism in the specific organs/tissues via the specific amino acid signaling pathway.

Elucidating the detailed molecular mechanisms of this amino acid signaling over metabolic regulation should give us a novel point of view about the adaptive response to protein-malnutrition or starvation. We are currently collecting data to enable us to define the underlying mechanisms by identifying candidates mediating this signaling with metabolomics and transcriptomics approaches. We believe that it should shed light on new possible nutritional/pharmacological treatments for NAFLD or on agricultural applications to control lipid-content of livestock products by means of the nutritional intervention.

## Materials and Methods

### Materials

For animal experimental diets, vitamin mixture, mineral mixture, cellulose powder and corn starch were purchased from Oriental Yeast (Tokyo, Japan), and soybean oil was from Nacalai Tesque (Tokyo, Japan). 10× Earle’s buffered salt solution (EBSS) and 100× MEM vitamin solution were purchased from Sigma Aldrich (St. Louis, MO, USA). Dulbecco’s modified Eagle’s medium (DMEM), phosphate-buffered saline (PBS), and Hanks’ buffered salt solution were purchased from Nissui Pharmaceutical CO., (Tokyo, Japan). Fetal bovine serum (FBS) and bovine insulin were obtained from Sigma Aldrich (St. Louis, MO, USA). Penicillin and streptomycin were obtained from Banyu Pharmaceutical CO., (Ibaraki, Japan). Anti-β-actin antibody was obtained from Santa Cruz Biotechnology, Inc. (Santa Cruz, CA USA). Anti-phospho-Akt (Ser-473) antibody, anti-Akt antibody, anti-phospho-Erk antibody and anti-Erk antibody were obtained from Cell Signaling Technology, Inc. (Danvers, MA, USA). Horseradish peroxidase (HRP)-conjugated secondary anti-rabbit and anti-mouse IgG antibody were obtained from GE Healthcare (Pittsburgh, PA, USA). Radiolabeled substances were purchased from PerkinElmer Life Science (Boston, MA, USA). Other chemicals were of the reagent grade available commercially.

### Isolation and primary culture of rat hepatocytes

Hepatocytes were prepared as described previously^34^. After 24-hour-culture in Williams’ E medium, cells were subjected to each experiment.

### Cell culture and cell experiments

H4IIE-C3 cells (a rat hepatoma cell line, ATCC CRL-1600) and HuH7 cells (a human hepatoma cell line, JCRB0403) were grown in a Dulbecco’s modified Eagle medium (DMEM) supplemented with 10% FBS and antibiotics under 5% CO_2_ at 37 °C. Reaching sub-confluency, media were changed to experimental media (Supplementary Table 1), then cultured for respective time.

To count the number of cells, the culture medium was removed and trypsin solution [2.5 g/l trypsin, 0.2 g/l EDTA, antibiotics] was overlaid. Dispersing the cells singly, the numbers were counted manually using hemocytometer.

To quantify the amount of genomic DNA, cells were lysed with lysis buffer [1.21 g/l Tris-HCl (pH8.0), 8.78 g/l NaCl, 3.72 g/l EDTA, 0.5% SDS], followed by proteinase K, RNase A and phenol/chloroform treatment. After precipitating the DNA with isopropanol, it was reconstituted in DW to be measured its concentration.

### Lipid synthesis assay

Hepatocytes were grown in 10%FBS/DMEM on collagen-coated 24-well plates (IWAKI, Japan) to reach sub-confluency. On the experiment, media were changed to Full or Zero medium containing 3 µCi/ml D-[^14^C(U)]-glucose (PerkinElmer Japan) and cells were incubated for another several hours indicated in Fig. 2. Then cells were lysed with 200 µl/well of 0.2 N NaOH and the lysates were subjected to sequential biphasic separation in which they were mixed well with 800 µl methanol:chloroform solution (1:1, v/v) and 200 µl saturated NaCl solution followed by centrifugation at 13,000 × g, 4 °C for 5 minutes. The organic (chloroform) layer was transferred into 5 ml of Clearsol II (Nacalai Tesque, Japan) to measure the ^14^C-radioactivity by Liquid Scintillation Counter (LSC6100, ALOKA).

### Glucose uptake assay

The protocol was described previously^34^.

### Protein extraction from cultured cells and immunoblotting

The protocol was described previously^34^.

### Animals

5-week-old male Wistar rats were purchased from Charles River Japan (Kanagawa, Japan). The rats were caged individually and kept in a room maintained at 24 ± 1 °C with 50–60% humidity and a 12 h-light (8:00–20:00)/12 h-dark (20:00–8:00) cycle. They were allowed free access to food and water during the experiment.

When the animal experiments were carried out, all rats were fed a normal chow for 3 days at first and control experimental diet containing 15% (w/w) amino acids (detailed composition is shown in Supplementary Table 2) for 4 next days as a training, then divided into some experimental groups. Thereafter, each group was given for 7 days either control diet or each experimental diet in which total amino acids or only a specific amino acid was restricted to 1/3 of control diet (Supplementary Table 2). All the diets were prepared by ourselves. In the experimental period, body weight and food intake of all rats were measured at 10:00 a.m. every day. In collecting the tissues, rats were anesthetized with isoflurane (DS Pharma Animal Health, Japan) after one-hour fasting and then blood from the carotid artery and the livers of each rat were obtained.

All animal care and experiments conformed to the Guidelines for Animal Experiments of The University of Tokyo and were approved by the Animal Research Committee of The University of Tokyo.

### Measurement of liver/cellular TG/Cholesterol

Total lipids in the liver were extracted according to the Folch’s method^35^ with modification, in which the frozen liver was homogenized with methanol:chloroform solution (1:2, v/v) followed by the addition of one fifth times of 0.8% KCl and centrifugation at 13,000xg, 4 °C for 10 minutes. Then organic (chloroform) layer was collected, whose solvent was evaporated and the left lipids were reconstituted in isopropanol. In the same way, lipids in cultured cells were extracted with methanol:chloroform solution (1:1, v/v) and saturated NaCl solution. TG and cholesterol content in the lipid extract was measured using Triglyceride E-test Wako (WAKO, Japan) and Cholesterol E-test Wako (WAKO, Japan) respectively.

### Analysis of serum amino acids

Each serum was deproteinized by adding the same volume of 3% trichloroacetic acid solution. After 10,000xg centrifugation for 30 minutes, the supernatant was applied to high speed amino acid analyzer (L-8900, HITACHI, Japan), according to the manufacturer’s instruction.

### Machine learning analysis

SOM and MLP analysis was performed as previously described^15,17^. For more detailed information and concrete algorithm, please see the referenced reports^14,15,17^. In brief (Supplementary Fig. S4), in SOM analysis, an initial map was prepared first consisting of 30 × 30 units, each carrying a random vector. Euclidean distances between an input vector containing serum amino acid concentrations of a rat for training data and every units on a map were calculated. One unit with minimal distance was chosen and then vectors on it and its neighborhood units were modified to make them closer to the input vector, which is applied for all the input data. These operations were repeated >1000 times to obtain a final map. In MLP analysis, input vectors and output vectors were prepared first containing serum amino acid concentrations or liver TG levels of rats for training data respectively. Input vectors were mapped to the output space via 3–5 spaces connected with each other with non-linear functions as an initial step and error rate between mapped values and actual measured liver TG values were calculated. The functions were then modified to minimize the error rate and these operations were repeated 1000 times to obtain an optimized functions. MLP setting was following; Loss function, MSE: Gradient method, RMSprop (Learning coefficient = 0.001: Weight decay coefficient = 0.9): Activation function, ReLU: Dropout = 0.5: Number of learning = 1000. In both SOM and MLP analysis, finally obtained map or function were applied for the data of rats for test data (“unlearned rats”) to evaluate their reliability.

### Statistical analysis

Data are expressed as means ± S.E.M. Comparisons between two groups were performed using Student’s t-test. Comparisons among more than two groups were carried out by one-way or two-way ANOVA. If the P-value obtained from ANOVA was under 0.05, the Tukey-Kramer’s post hoc test was performed. Values of *P* < 0.05 were considered to be statistically significant.

## Electronic supplementary material

Supplementary information
