# Supplementary material for: Importance of Serum Amino Acid Profile for Induction of Hepatic Steatosis under Protein Malnutrition
Source: Sci Rep. 2018 Apr 3;8:5461. doi: 10.1038/s41598-018-23640-8 (PMC5882898; doi:10.1038/s41598-018-23640-8)
Supplement: Supplementary file 1 — Supplementary information [file 41598_2018_23640_MOESM1_ESM.pdf]

# Importance of Serum Amino Acid Profile for Induction of Hepatic Steatosis under Protein Malnutrition

Hiroki Nishi[1], Daisuke Yamanaka[2], Hiroyasu Kamei[1], Yuki Goda[1], Mikako Kumano[1], Yuka Toyoshima[3], Asako Takenaka[4], Masato Masuda[5], Yasushi Nakabayashi[5], Ryuji Shioya[5], Naoyuki Kataoka[1], Fumihiko Hakuno[1]*, Shin-Ichiro Takahashi[1+]

[1] Departments of Animal Sciences and Applied Biological Chemistry, [2] Department of Veterinary Medical Sciences, Graduate School of Agriculture and Life Sciences, The University of Tokyo, Tokyo, Japan

[3] Department of Bioregulation, Nippon Medical School, Kanagawa, Japan

[4] Department of Agricultural Chemistry, School of Agriculture, Meiji University, Kanagawa, Japan

[5] Center for Computational Mechanics Research, Toyo University, Kawagoe, Japan

[+] **Corresponding author:** Shin-Ichiro Takahashi, Ph.D., Departments of Animal Sciences and Applied Biological Chemistry, Graduate School of Agriculture and Life Sciences, The University of Tokyo, 1-1-1 Yayoi, Bunkyo-ku, Tokyo, Japan

TEL: +81-3-5841-1310; FAX: +81-3-5841-1311;

E-mail: atkshin@mail.ecc.u-tokyo.ac.jp

* **Co-corresponding author:** Fumihiko Hakuno, Ph.D., Departments of Animal Sciences and Applied Biological Chemistry, Graduate School of Agriculture and Life Sciences, The University of Tokyo, 1-1-1 Yayoi, Bunkyo-ku, Tokyo, Japan

TEL: +81-3-5841-1310; FAX: +81-3-5841-1311;

E-mail: ahakuno@mail.ecc.u-tokyo.ac.jp

## Supplementary Tables

### Supplementary Table 1. Compositions of experimental media

| | Full | Zero | ΔGly | ΔAla | … |
|---|---|---|---|---|---|
| Glycine | 30.0 | 0 | 0 | 30.0 | |
| L-Alanine | 35.6 | 0 | 35.6 | 0 | |
| L-Serine | 42.0 | 0 | 42.0 | 42.0 | |
| L-Threonine | 95.0 | 0 | 95.0 | 95.0 | |
| L-Cystine | 48.0 | 0 | 48.0 | 48.0 | |
| L-Methionine | 30.0 | 0 | 30.0 | 30.0 | |
| L-Glutamine | 584.0 | 0 | 584.0 | 584.0 | |
| L-Asparagine・$H_2O$ | 60.0 | 0 | 60.0 | 60.0 | |
| L-Glutamic acid | 58.8 | 0 | 58.8 | 58.8 | |
| L-Aspartic acid | 53.2 | 0 | 53.2 | 53.2 | |
| L-Valine | 94.0 | 0 | 94.0 | 94.0 | |
| L-Leucine | 105.0 | 0 | 105.0 | 105.0 | |
| L-Isoleucine | 105.0 | 0 | 105.0 | 105.0 | |
| L-Phenylalanine | 66.0 | 0 | 66.0 | 66.0 | |
| L-Tyrosine | 72.4 | 0 | 72.4 | 72.4 | |
| L-Tryptophan | 16.0 | 0 | 16.0 | 16.0 | |
| L-Lysine・HCl | 146.0 | 0 | 146.0 | 146.0 | |
| L-Arginine・HCl | 84.0 | 0 | 84.0 | 84.0 | |
| L-Histidine | 31.0 | 0 | 31.0 | 31.0 | |
| L-Proline | 46.0 | 0 | 46.0 | 46.0 | |
| EBSS | 10% | 10% | 10% | 10% | |
| vitamin solution | 1% | 1% | 1% | 1% | |
| $NaHCO_3$ | 2.2 | 2.2 | 2.2 | 2.2 | |
| D-glucose | 4.5 | 4.5 | 4.5 | 4.5 | |

[mg/L]

## Supplementary Table 2. Compositions of experimental diets

| | CN | 5AA | ΔIle | ΔLeu | … | 5AA+Suf.E | 5AA+R | 5AA+T |
|---|---|---|---|---|---|---|---|---|
| L-Isoleucine | 7.1 | 2.4 | 2.4 | 7.1 | | 2.4 | 2.4 | 2.4 |
| L-Leucine | 13.0 | 4.3 | 13.0 | 4.3 | | 4.3 | 4.3 | 4.3 |
| L-Lysine・HCl | 14.1 | 4.7 | 14.1 | 14.1 | | 4.7 | 4.7 | 4.7 |
| DL-Methionine | 6.4 | 2.1 | 6.4 | 6.4 | | 2.1 | 2.1 | 2.1 |
| L-Cystine | 0.8 | 0.3 | 0.8 | 0.8 | | 0.3 | 0.3 | 0.3 |
| L-Phenylalanine | 7.2 | 2.4 | 7.2 | 7.2 | | 2.4 | 2.4 | 2.4 |
| L-Tyrosine | 7.8 | 2.6 | 7.7 | 7.8 | | 2.6 | 2.6 | 2.6 |
| L-Threonine | 6.1 | 2.0 | 6.1 | 6.1 | | 2.0 | 2.0 | 6.1 |
| L-Tryptophan | 1.7 | 0.6 | 1.7 | 1.7 | | 0.6 | 0.6 | 0.6 |
| L-Valine | 9.2 | 3.1 | 9.1 | 9.2 | | 3.1 | 3.1 | 3.1 |
| L-Histidine | 4.1 | 1.3 | 4.1 | 4.1 | | 1.3 | 1.3 | 1.3 |
| L-Arginine | 5.2 | 1.7 | 5.2 | 5.2 | | 1.7 | 5.2 | 1.7 |
| L-Alanine | 4.1 | 1.3 | 4.1 | 4.1 | | 1.3 | 1.3 | 1.3 |
| L-Aspartic acid | 5.1 | 1.7 | 5.1 | 5.1 | | 1.7 | 1.7 | 1.7 |
| L-Asparagine・$H_2O$ | 5.8 | 1.9 | 5.7 | 5.8 | | 1.9 | 1.9 | 1.9 |
| L-Glutamic acid | 14.6 | 4.9 | 14.7 | 14.6 | | 115.0 | 4.9 | 4.9 |
| Glycine | 2.6 | 0.9 | 2.6 | 2.6 | | 0.9 | 0.9 | 0.9 |
| L-Proline | 15.0 | 5.0 | 15.0 | 15.0 | | 5.0 | 5.0 | 5.0 |
| L-Serine | 8.1 | 2.7 | 8.1 | 8.1 | | 2.7 | 2.7 | 2.7 |
| L-Glutamine | 14.6 | 4.9 | 14.7 | 14.6 | | 4.9 | 4.9 | 4.9 |
| cellulose | 100 | 100 | 100 | 100 | | 100 | 100 | 100 |
| vitamin mixture | 10 | 10 | 10 | 10 | | 10 | 10 | 10 |
| mineral mixtue | 40 | 40 | 40 | 40 | | 40 | 40 | 40 |
| soybean oil | 50 | 50 | 50 | 50 | | 50 | 50 | 50 |
| corn starch | 647.5 | 749.2 | 652.2 | 656.2 | | 639.1 | 745.7 | 745.1 |
| total | 1000 | 1000 | 1000 | 1000 | | 1000 | 1000 | 1000 |

[g/kg]

## Supplementary Figures

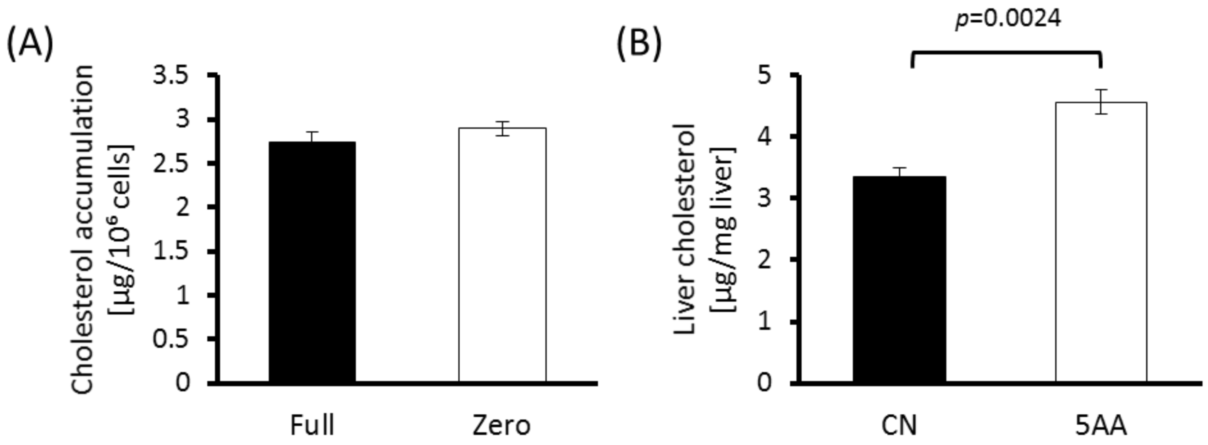


**Supplementary Fig. S1 Cholesterol levels in response to amino acid deficiency in H4IIE cells and the rat liver.**
(A) H4IIE cells were cultured in Full or Zero medium for 24 hours and then intracellular cholesterol level was measured. (B) Six-week-old male Wistar rats were fed CN or 5AA diet ad libitum for seven days and their livers were collected. Total lipids were extracted from the liver and cholesterol levels were measured. bar; means±S.E. (A: n=3, B: n=5)

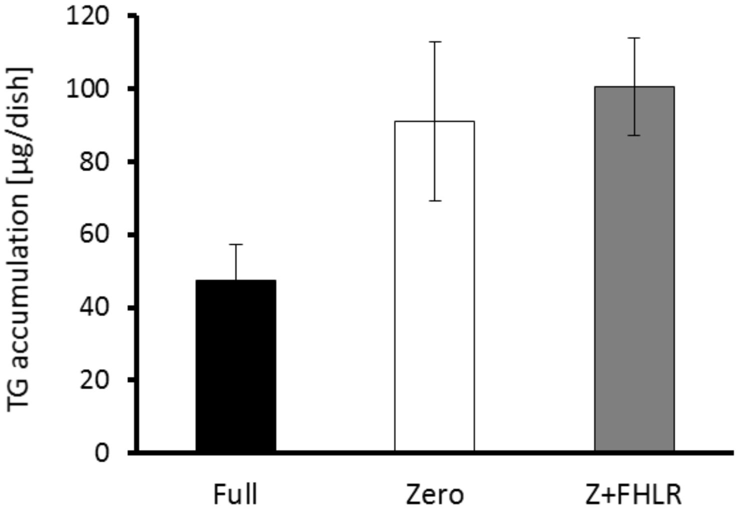


**Supplementary Fig. S2 An effect of Phe, His, Leu and Arg supplementation on TG accumulation in H4IIE cells.**
H4IIE cells were cultured for 24 hours in Full, Zero or Zero medium supplemented with Phe, His, Leu and Arg at the same concentration contained in Full medium and then intracellular TG level was measured. bar; means±S.E. (n=3)

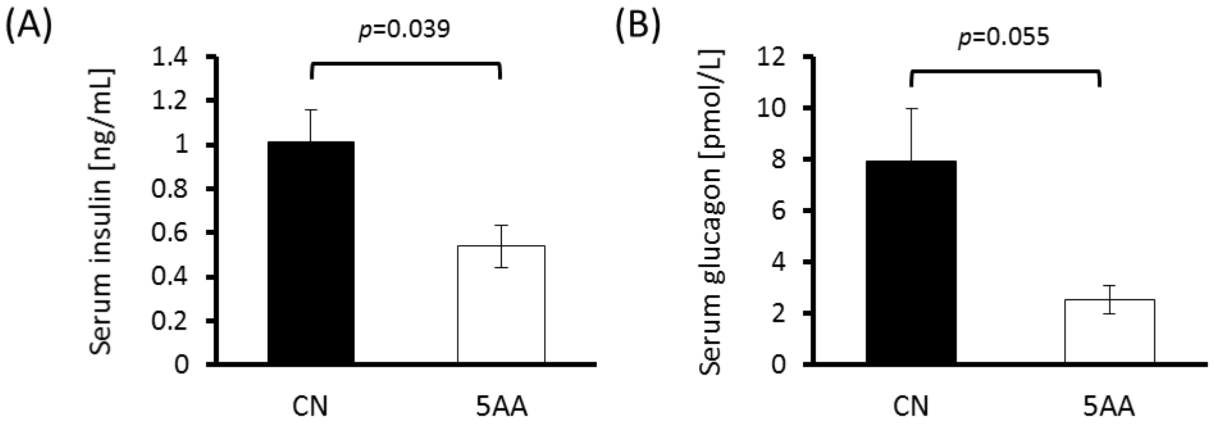


**Supplementary Fig. S3 Serum insulin and glucagon levels in low-amino acid diet-fed rats**
Six-week-old male Wistar rats were fed CN or 5AA diet ad libitum for seven days and their sera were collected. Serum insulin (A) and glucagon (B) levels were measured.
bar; means±S.E. (n=5)

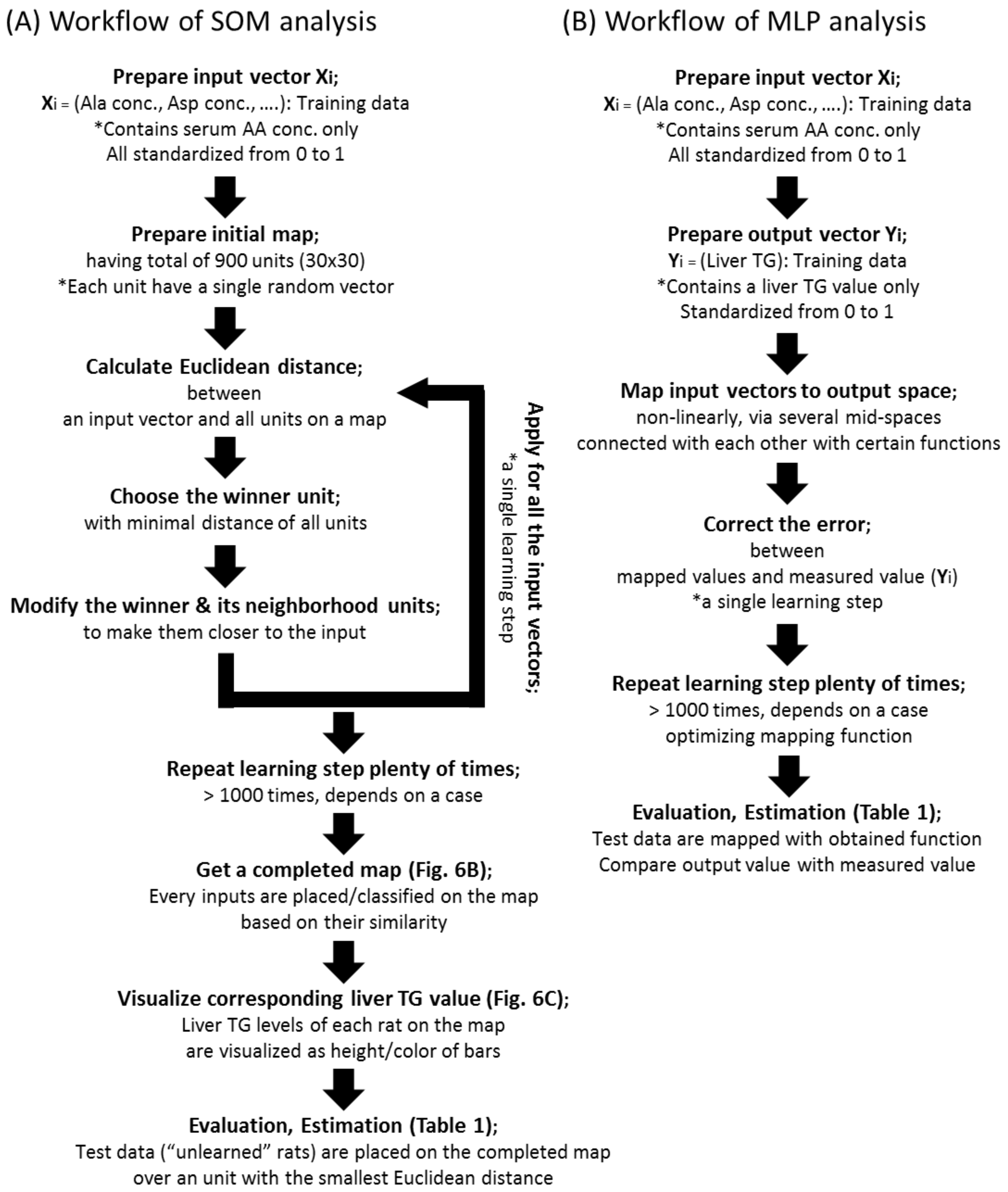


**Supplementary Fig. S4 Flowcharts of machine learning.**
Workflows of SOM (A) and MLP (B) analysis are shown briefly as a flowchart.

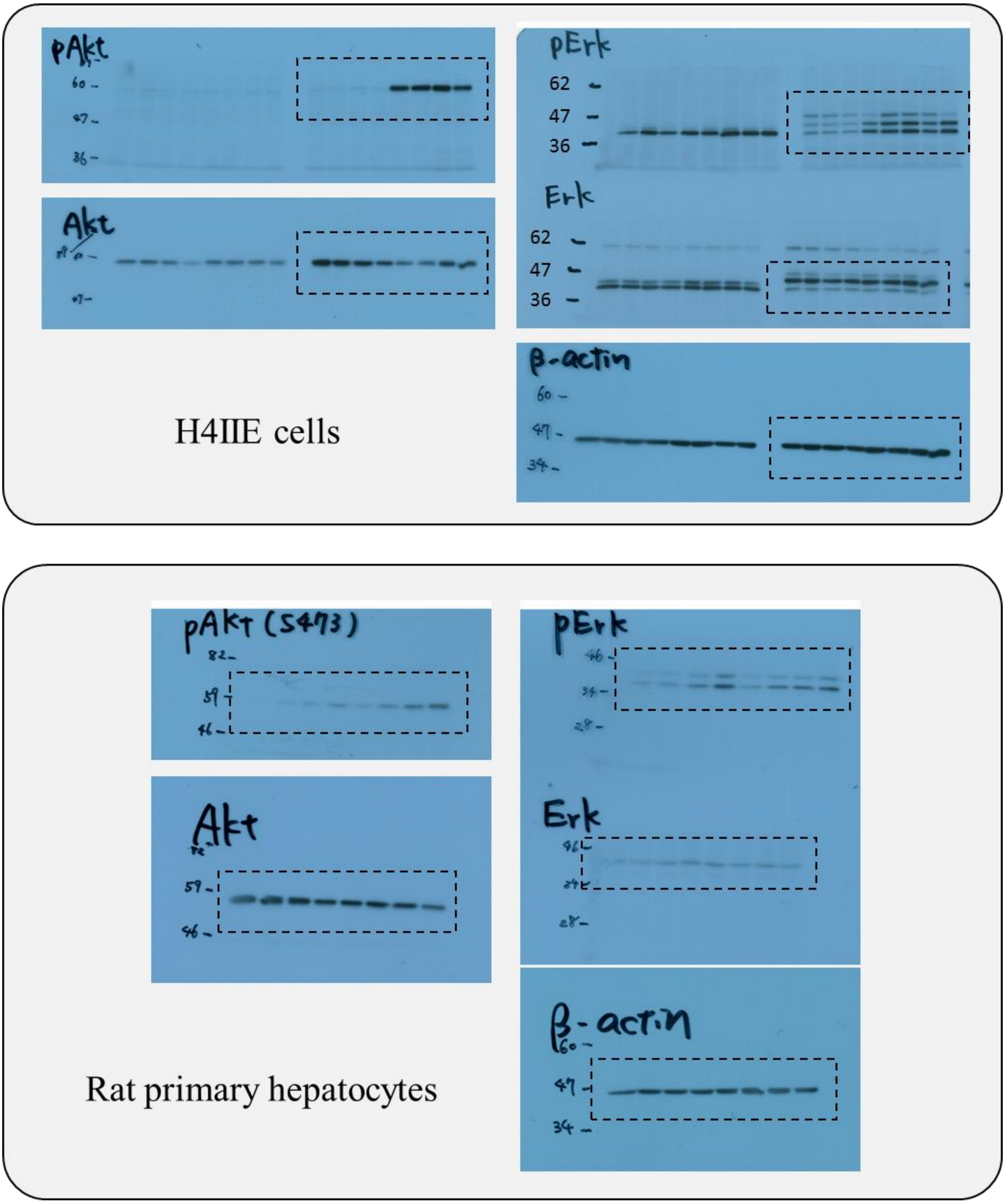


**Supplementary Fig. S5 Unprocessed scans of blots shown in Fig. 3 (B) and (D).**
Dotted rectangles delimit cropped areas used in Fig. 3.

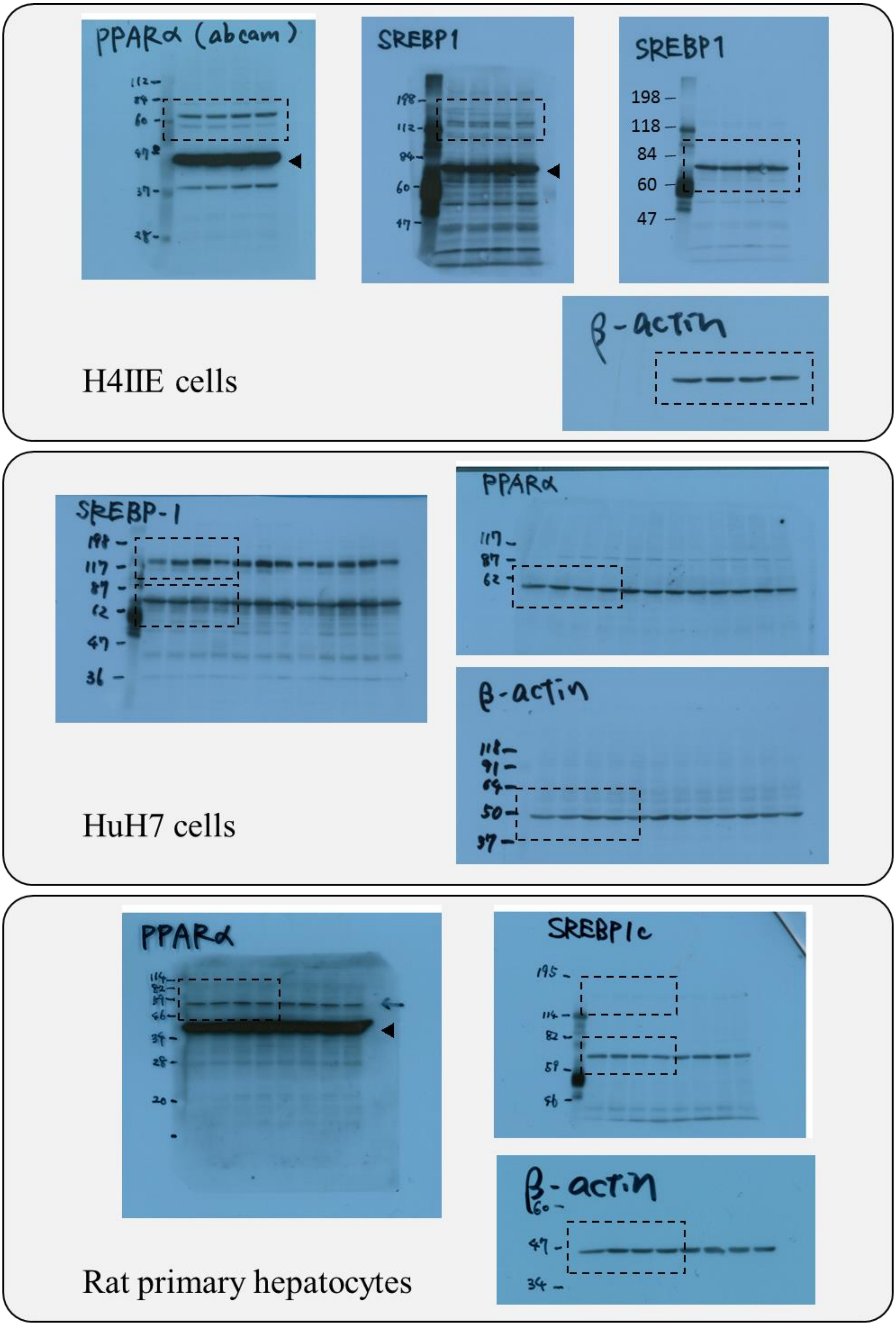


**Supplementary Fig. S6 Unprocessed scans of blots shown in Fig. 3 (F).**
Dotted rectangles delimit cropped areas used in Fig. 3. Arrowheads indicate non-specific bands.
